# Minimization of Adverse Effects Associated with Dental Alloys

**DOI:** 10.3390/ma15217476

**Published:** 2022-10-25

**Authors:** Marianna Arakelyan, Gianrico Spagnuolo, Flavia Iaculli, Natalya Dikopova, Artem Antoshin, Peter Timashev, Anna Turkina

**Affiliations:** 1 Therapeutic Dentistry Department, Institute for Dentistry, Sechenov University, 119991 Moscow, Russia; 2 Department of Neurosciences, Reproductive and Odontostomatological Sciences, University of Naples “Federico II”, 80138 Napoli, Italy; 3 Institute for Regenerative Medicine, Sechenov University, 119991 Moscow, Russia; 4 World-Class Research Center “Digital Biodesign and Personalized Healthcare”, Sechenov University, 119991 Moscow, Russia; 5 Chemistry Department, Lomonosov Moscow State University, 119234 Moscow, Russia

**Keywords:** dental alloys side effects, oral galvanism, dental alloys biocompatibility, corrosion, tribocorrosion, hypersensitivity to metals

## Abstract

Metal alloys are one of the most popular materials used in current dental practice. In the oral cavity, metal structures are exposed to various mechanical and chemical factors. Consequently, metal ions are released into the oral fluid, which may negatively affect the surrounding tissues and even internal organs. Adverse effects associated with metallic oral appliances may have various local and systemic manifestations, such as mouth burning, potentially malignant oral lesions, and local or systemic hypersensitivity. However, clear diagnostic criteria and treatment guidelines for adverse effects associated with dental alloys have not been developed yet. The present comprehensive literature review aims (1) to summarize the current information related to possible side effects of metallic oral appliances; (2) to analyze the risk factors aggravating the negative effects of dental alloys; and (3) to develop recommendations for diagnosis, management, and prevention of pathological conditions associated with metallic oral appliances.

## 1. Introduction

Metal alloys are one of the most popular materials used in current dental practice. Metals are used in the fabrication of artificial crowns and bridges, prefabricated posts and cast post-and-core inlays, clasp removable dentures and dental implants, as well as fixed orthodontic equipment. More than half of Europeans have at least one metal structure in their oral cavity, such as an artificial crown, inlay, or dental bridge [1]. In the last 20 years, there has been a steady upward trend in the number of people having dental implants. For example, in the USA, the proportion of people with at least one dental implant increased from 0.7% to 5.7% between 2000 and 2016. According to the experts, by 2026, the proportion of people with dental implants may reach 23% [2]. Moreover, the use of fixed orthodontic appliances is also predicted to rise, including in the adult category [3]. The most commonly used metal alloys in dentistry include cobalt-chromium, nickel-titanium, nickel-chromium, and titanium alloys.

In some cases, the interaction of dental alloys with the environment of the oral cavity may cause undesirable phenomena that not only worsen the quality of life of the patient but also may negatively affect oral tissues [4,5] or even the whole organism [6]. 

The present comprehensive literature review aims (1) to summarize the current information related to possible side effects of metallic oral appliances; (2) to analyze the risk factors aggravating the negative effects of dental alloys; and (3) to develop recommendations for diagnosis, management, and prevention of pathological conditions associated with metallic oral appliances.

## 2. The Main Effects of Dental Alloys on the Human Organism

**Figure 1:**
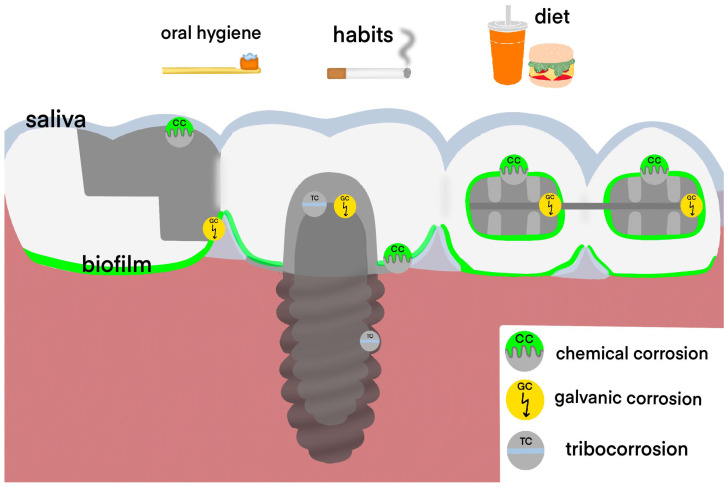
Interactions of dental alloys with the oral environment. The dental alloys are affected by intraoral (saliva, oral biofilm) and extraoral (food, drinks, oral hygiene, etc.) factors, which lead to chemical corrosion. Moreover, metallic appliances produced from different dental alloys in the oral environment may have galvanic interaction. In addition to that, the components of dental implants form a tribosystem where corrosion and wear are combined.

In the oral cavity, the metallic structures are exposed to various extra-oral and intra-oral factors, which may cause their corrosion (Figure 1). Chemical corrosion is associated with saliva, exposure to acids from sour food and drinks, and exposure to acids produced by oral biofilm [7]. Moreover, the metallic appliances are affected by mastication load and oral hygiene products, which lead to mechanical-chemical corrosion [8]. The friction and wear of two contacting metallic appliances in a corrosive media is termed tribocorrosion [9]. Tribocorrosion may cause degradation of dental implants, which are usually composed of several metallic structures [10]. Galvanic corrosion of dental alloys occurs when two metallic appliances with different electrochemical potentials interact in the electrolytic medium (saliva) [11]. As a result of the corrosion, metal ions are released into the saliva and adjacent oral mucosa [12].

**Figure 2:**
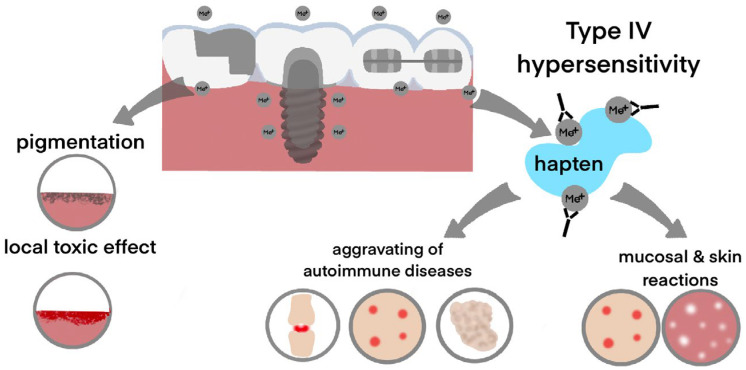
Local and systemic effects of dental alloys. Metal ions released from the dental alloys accumulate in the oral mucosa, causing its pigmentation or chronic inflammatory reactions. In allergic patients, metal ions may cause Type IV hypersensitivity, mostly affecting skin and oral mucosa. Moreover, hypersensitivity to metals may aggravate autoimmune diseases affecting joints, skin, and salivary glands.

The main effects of metal ions on the human organism are shown in Figure 2. The first effect is local cytotoxity. It has been shown that ions released from dental alloys may negatively affect periodontal fibroblasts [13,14] and epithelial cells [15]. The main mechanisms of metal cytotoxity are apoptosis [16] and oxidative stress [17,18], which may increase the risk of potentially malignant oral lesions [19]. Moreover, metal ions, including Ti^4+^, may stimulate the release of inflammatory mediators and tumor necrosis factor, causing chronic inflammation of the oral mucosa and peri-implant tissues [20,21,22]. Indeed, several in vivo and in vitro studies demonstrated that ions released from dental alloys could damage the DNA of oral mucosa cells [23,24,25,26]. However, other researchers revealed no genotoxic effect of Ti-based dental alloys and orthodontic alloys [27,28,29].

The second effect is allergic reactions. Metal ions may interact with human proteins as haptens and initiate hypersensitivity reactions [6,30]. The pathogenesis of the allergic reaction may be different, but the most common is a type IV reaction manifesting as chronic inflammation with lymphoid infiltration [31]. The corresponding immune response may develop in the form of local conditions such as lichenoid reaction [32] or may involve other tissues and organs [33,34].

Moreover, metal ions are swallowed together with saliva and, potentially, may accumulate in different tissues and organs [35]. However, the systemic toxic effect of dental alloys is doubtful as the daily intake of metal ions from intraoral appliances is much below the toxic limit value [36,37,38].

Another significant problem is galvanic current that occurs when dissimilar metals are present in the oral cavity with saliva serving as an electrolyte [39]. Back in 1984, S. Ayers mentioned that the direct current below the level of 10 µA is not critical [40]. According to von Fraunhofer et al., a galvanic current of 20 µA is associated with a pain reaction [41]. The current from 75 µA to 100 µA may cause chronic irritation of oral mucosa [40]. Chronic electrical trauma of the oral mucosa is considered a risk factor for different oral lesions, including potentially malignant lesions such as verrucous leukoplakia and oral lichen planus [11]. For example, Wartenberg et al. applied direct current to the oral carcinoma cell culture and observed apoptosis [42], while Korrah et al. on leukoplakia cells showed that the direct current may cause effects similar to those in the case of malignization [43].

However, in the oral cavity, it is difficult to clarify the leading pathogenesis mechanism since a synergistic effect of two or even three factors may be observed.

## 3. Clinical Manifestations of Adverse Effects Associated with Dental Alloys

Adverse events associated with dental alloys can be conditionally divided into local and systemic ones. In addition, special attention should be paid to the potential relationship between the presence of metal structures in the oral cavity and the development of systemic diseases.

### 3.1. Local Manifestations

**Figure 3:**
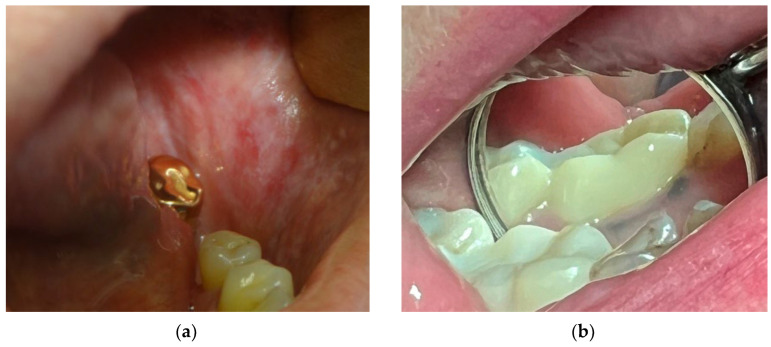

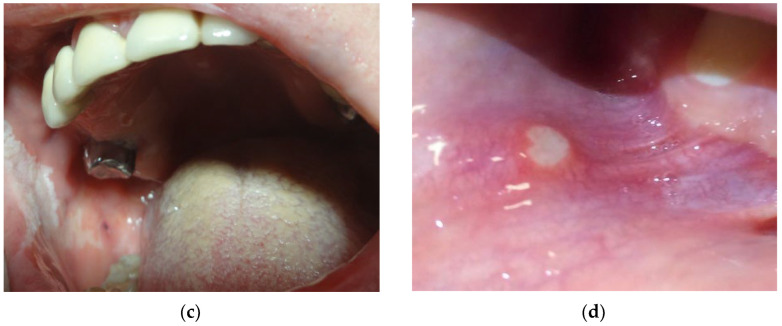
The most common oral lesions that may be associated with dental alloys: (**a**)—oral lichen planus; (**b**)—oral mucosa pigmentation; (**c**)—oral leukoplakia; (**d**)—recurrent aphthous stomatitis.

The most common local reactions are as follows:

- mouth burning without any visible lesions of the oral mucosa is commonly associated with galvanic current between different dental alloys. According to different authors, in patients with metal structures in the mouth, a burning sensation was observed with a frequency of 17% to 33% [39,44,45,46]. However, it should be kept in mind that burning mouth syndrome may develop in patients without metallic appliances and may be associated with a number of other systemic and local factors, such as vitamin deficiency, hormonal changes associated with menopause, local infections of the oral cavity, xerostomia, denture-related lesions, allergies, medications, and systemic diseases, including diabetes mellitus [47].
- oral lichen planus and lichenoid reaction (Figure 3a) manifest as multiple white papules, merging into the characteristic Wickham rete [48]. According to different authors, these phenomena are observed with a frequency of 12% to 78% among patients with metal structures in the mouth [44,49,50]. The condition may develop due to chronic irritation by galvanic current or as a delayed-type hypersensitivity reaction. Lichenoid reactions of the oral cavity are histologically or clinically indistinguishable from lichen planus, even though the latter may present within skin lesions and is not necessarily localized in direct contact with a metal structure. Both lichenoid reactions and lichen planus are precancerous [51].
- pigmentation of oral mucosa (Figure 3b) appears as a dark spot on the mucosa near to the metal structure, and it most often occurs upon contact with amalgam and silver-containing alloys. Moreover, metal particles can deposit on the oral mucosa during the placement or removal of amalgam fillings and appear as dark pigmented lesions [52]. In the presence of a galvanic couple, the pigmentation processes can be enhanced [53]. However, it must be taken into account that any dark pigmented lesion can be not only a benign discoloration but also potentially represent melanoma [54,55,56,57].
- leukoplakia (Figure 3c) is characterized by the emergence of increased keratinization areas on the mucous membrane. The prevalence of leukoplakia ranges from 0.5 to 3.4% and occurs most often in people older than 50 years [58]. It should be noted that the frequency of malignant transformation of leukoplakia ranges from 0.1 to 17% [59]. According to the observations of Gönen Z.B. et al., hyperkeratotic lesions may occur due to a hypersensitivity reaction to amalgam [58].
- erosive and ulcerative lesions (Figure 3d) are mostly the form of allergic reactions to metals and manifest as recurrent aphthous stomatitis [60].

### 3.2. Systemic Adverse Effects of Dental Alloys

Commonly, systemic adverse effects of dental alloys are associated with hypersensitivity reactions, which may include typical clinical manifestations such as dermatitis, itching, eczema, and Quincke’s edema [33,61]. Moreover, chronic fatigue syndrome, headaches, and polyarthritis were observed [62,63]. An interesting case report of allergic gastritis associated with dental alloys was published by Pföhler et al. in 2016 [64]. In 2020, Zigante et al. examined 228 patients with fixed orthodontic appliances. Hypersensitivity to titanium and nickel was diagnosed in 16% of study participants. The symptoms associated with hypersensitivity were as follows: hypogeusia, hyposmia, tongue or face edema, and watering [65]. 

### 3.3. Associations between Dental Alloys and Systemic Diseases

Potential risks associated with dental amalgam are widely discussed in the literature. Mercury may cause oxidative stress, damage mitochondria and lipid membranes, change DNA structure and stimulate autoimmunity [66], which potentially may increase the risk of neurodegenerative diseases. Within the last 20 years, clinical studies evaluated possible associations of dental amalgam with neurological and autoimmune diseases and showed controversial results [67,68,69,70,71]. According to the systematic review published by Gallusi et al. in 2021, amalgam restorations are not associated with an increased risk of any systemic disease [72]. Nevertheless, the issue of the potential toxicity of mercury released from dental amalgams is a matter of debate in the scientific literature [73].

The new diagnostic methods for metal hypersensitivity detection allowed the researchers to explore the role of dental alloys in the pathogenesis of autoimmune diseases. Several studies have shown an association between sensitivity to one or more metals and the severity of different autoimmune diseases [74,75,76,77]. In contrast, clinical studies demonstrated that metallic oral appliances did not increase the risk of autoimmune diseases [78,79,80].

However, it should be stressed that metallic intra-oral appliances are not always associated with any side effects, even in the case of galvanic coupling of dental alloys [81] or even if the patients are sensitive to metals [82]. It may be hypothesized that clinical reaction to dental alloys strongly depends on the patient’s individual characteristics such as composition and possible galvanic coupling of dental alloys, saliva properties, oral hygiene, dietary behaviors, systemic diseases, etc. Risk factors aggravating dental alloy side effects are further discussed below.

## 4. Factors Affecting the Risk of Dental Alloys Side Effects

Factors which may aggravate the negative effects of metallic oral appliances are listed in Table 1. 

### 4.1. Corrosion Resistance and Biocompatability of Common Dental Alloys

Among all dental alloys, dental amalgam and nickel-chromium alloys are supposed to have the least corrosion resistance both in the artificial saliva and in the acidic environment [83,84]. The common amount of absorbed mercury from amalgam restoration is reported to be less than 5 µg per day [73], but the levels of mercury accumulation and excretion are determined genetically and may significantly differ between individuals [85]. The release of nickel ions may reach 5.22 µg/cm^2^/day from nickel-chromium alloy [38] and 0.93 µg/day from orthodontic archwires [86]. Cobalt-chromium alloys are more resistant to corrosion, as it was shown by Kaasapidou et al., the Co ion release per 7 days did not exceed 2.6 µg/cm^2^ [87]. Pure titanium and titanium alloys are commonly used for dental implants. In general, titanium is resistant to corrosion due to the formation of titanium dioxide film on its surface [88], but dental implants may release titanium in the form of nanoparticles both within the implantation procedure and after prolonged interaction with the oral environment [21,22]. To reduce corrosion, titanium may be alloyed with Al, V, Zr, Ta, Mo, or Cr [89]. Titanium alloys are more corrosion resistant compared to pure titanium [90], and the release of Ti from TiAlV alloy was shown to be 16 ng/cm^2^/day [91]. Moreover, it should be noted that Ti alloy may become the source of V and Al ions, which are potentially toxic [91,92]. 

The galvanic current depends on the potential differences between dental alloys. According to the multiple in vitro studies, significant galvanic current may occur in the following galvanic couples: dental amalgam and cobalt-chromium alloy [93,94]; titanium alloy and dental amalgam [95]; nitinol archwire and titanium brackets [96]; nitinol archwire and iron brackets [96,97]; titanium dental implant and cobalt-chromium or nickel-chromium alloys [98,99,100,101]. Moreover, the surface area of metallic appliances should be taken into account. For instance, Nayak et al. in 2015 showed that the current increased together with increasing of cathode surface area and decreasing of anode surface area [97]. 

Regarding the tribocorrosion, the titanium–titanium combination showed more wear than titanium-zirkonia and titanium-Roxolid [102]. 

It should be noted that the data related to corrosion of dental alloys are mainly obtained in experimental studies, which cannot always be clinically proven. The standard methods for corrosion assessment are recommended by ISO 10271:2020 and include static immersion tests with lactic acid, sulfide tarnish static/cyclic immersion tests, and electrochemical tests [103]. However, many studies use artificial saliva with additional components such as fluoride, lisozyme or urea [83]. In addition, experimental models reproducing saliva flow [38] or tribocorrosion [104] were introduced.

As for the cytotoxicity of common ions released from dental alloys, Ni, Zn, and Cu ions were defined as the most toxic elements, while Fe, Cr, Mo, Al, and Pd ions showed less cytotoxic effects [20,105,106]. Ti is supposed to be much more biocompatible than other metals, however, Ti ions released from dental implants may cause periimplantitis or type IV allergic reactions [107]. Evaluation of dental alloy biocompatibility is standardized by ISO 7405:2018 which refers to in-vitro cytotoxicity assessment with cell culture (direct contact tests or extract tests) [108]. To close the experimental conditions to a real clinical situation, new tissue models were developed. Commercially available three-dimensional models of keratinized and non-keratinized oral epithelium are suitable for evaluation of dental materials’ effects on the oral mucosa [109]. Oral mucosa-on-a-chip is a complex model that allows assessing the effects of dental materials on different layers of the oral mucosa, including keratinocytes, fibroblasts, and collagen [110]. Moreover, recently, the new multi-organ-on-chip model was developed for the evaluation of the systemic toxicity of dental alloys [111]. The future use of standardized tissue models will ensure accurate and reproducible results related to local and systemic effects of dental alloys. 

### 4.2. Composition and pH of the Saliva 

Saliva plays a crucial role in the surface degradation of metallic oral appliances. 

Saliva is an electrolytic medium in which normal electroconductivity varies from 3.5 mS/cm to 4.73 mS/cm [112,113,114]. However, pH, ionic composition, and, accordingly, electroconductivity of the whole saliva may significantly vary both between individuals and within individuals depending on the age [115], time of the day [116], and even on the menstrual cycle phase [117]. The decrease in salivary pH is the significant factor aggravating chemical corrosion and galvanic corrosion [100,118]. The decrease in salivary pH is commonly observed in patients with periodontal disease [119] and in patients with multiple caries [120]. Moreover, many systemic diseases and conditions may change the electrochemical characteristics of saliva [112,121,122,123,124,125,126].

It was also shown that an increased level of carbamide in saliva significantly enhances corrosion of dental alloys [127]. The effects of proteins are poorly investigated. On the one hand, proteins, especially mucine, form a protective film on the metallic surfaces and reduce tribocorrosion [128]. On the other hand, some proteins may stimulate corrosion, wherein different proteins even with similar amino acids may show different effects on dental alloys [129].

### 4.3. Oral Microbiota

Dental biofilm plays a significant role in the biodegradation of dental alloys. Acidogenic bacteria such as Streptococcus mutans, Lactobacillus reuteri, Streptococcus sanguis, Streptococcus mitis, Streptococcus sobrinus, Streptococcus salivarius, sulfate-reducing bacteria, sulfate-oxidizing bacteria, Veilonella, Actinomyces, and Candida albicans may aggravate corrosion of dental alloys [7,130,131]. Lipopolysaccharide of microbial origin dramatically increases tribocorrosion of dental implants [132]. On the other hand, normal oral microbiota such as Streptococcus salivarius work as lubricant on the metal surface and reduce tribocorrosion of metallic appliances in the oral cavity [133]. 

### 4.4. Oral Care Products 

Toothpastes and mouthrinses play a central role in the chemo-mechanical wear of all dental materials, including dental alloys. According to experimental tests, abrasive toothpastes cause degradation of the dental alloy surface quality, which leads to increased ion release, although the surface roughness remains within the normal range [134,135,136,137]. It should be noted that increased fluoride concentration in the oral environment dramatically increases galvanic corrosion of dental alloys [134,138,139,140], therefore, patients with metallic appliances are not recommended to use oral care products with fluorides. It was shown that Listerine^®^ mouthwash may stimulate nickel ions release and change the surface properties of nickel-titanium orthodontic archwires [141,142]. Chlorhexidine may increase corrosion of orthodontic implants [143], although a similar effect for brackets was not observed [139]. Ethanol-containing mouthwashes are supposed to be the most aggressive to dental alloys due to increased galvanic corrosion [139,144]. 

It is noteworthy that saliva replacement products, permanently used by xerostomic patients, may also influence galvanic and corrosion processes in the oral cavity. Spirk et al. figured out that the electroconductivity of several saliva replacements was up to two times higher than natural saliva [113]. 

### 4.5. Dietary Behaviors

The acidity of common foods and beverages may potentially influence the corrosion of dental alloys. Shahabi et al. showed that frequent intake of acidic products may significantly increase the corrosion of orthodontic brackets [145]. Recently, the effect of brewed coffee may increase ion release from nickel titanium alloy due to the decreased pH of saliva mixed with coffee [146]. Conversely, Parenti et al. detected in vitro no effect of acidic beverages on the surface structure of nickel-titanium orthodontic archwires [147]. 

### 4.6. Bad Habits

Tobacco smoke damages oral mucosa cells [148], slows regeneration [149], and increases the risk of potentially malignant oral lesions [150,151]. Regular alcohol consumption also negatively affects oral mucosa cells [152] and may stimulate malignant transformation of oral lesions [153]. Therefore, it can be assumed, that smoking and alcohol intake would aggravate any side effects associated with dental alloys. Moreover, considering the effect of ethanol-based oral rinses on dental alloys [139,144], we may suppose that alcohol may increase corrosion and galvanic processes in the oral cavity. 

### 4.7. Systemic Diseases and Conditions

Post-menopausal women are often mentioned as a risk group for mouth burning. According to previously published data, for post-menopausal women, the following changes in the oral cavity are commonly observed: decreased salivation, decreased salivary pH, and increased Ca^2+^ concentration in the whole saliva [154,155]. Moreover, the oral mucous membrane becomes more sensitive to different irritants, and taste disorders and mouth burning are also common [156]. As a result, post-menopausal women may potentially be more sensitive to galvanic current and should be included in the risk group for dental alloy side effects.

Gastroesophageal reflux disease should also be mentioned as a significant factor of dental alloy biodegradation, both due to the regular drop of intra-oral pH and the change of ionic composition of the whole saliva [121,122].

Diabetes mellitus is commonly associated with increased sensitivity of oral mucosa due to peripheral and autonomic neuropathy [157], which may increase mouth burning in the case of galvanic coupling of dental alloys in the oral cavity. Another factor increasing galvanic corrosion of dental alloys in patients with diabetes mellitus is the decreased salivary pH [126,158].

Similar symptoms are observed in patients with thyroid hypofunction: decreased pH of the whole saliva [123], altered taste [159], and mouth burning [160].

Electroconductivity of whole saliva increases in patients with dehydration associated with renal diseases or increased physical activity [112].

As mentioned above, patients with autoimmune diseases often have hypersensitivity to metals. That is why metallic oral appliances may worsen the patient’s condition [77,161]. In addition, increased electroconductivity of the saliva and poor healing potential of the oral mucosa were shown in patients after radiotherapy [113].

## 5. Approaches to Reduce the Risk of Adverse Events Associated with Dental Alloys

### 5.1. Industrial Methods to Reduce Corrosion of Dental Alloys

Corrosion resistance of dental alloys is affected not only by composition but also by manufacturing method [87] and surface polishing [162]. Several methods have been suggested to improve the corrosion resistance of oral metallic appliances.

Chemical passivation aims to form a protective oxide layer on the appliance surface. It was shown that corrosion of passivated cobalt-chromium alloy was significantly lower compared to non-passivated samples [163]. Citric acid passivation of dental implants may also be useful to minimize corrosion [164]. Another method to obtain a thick and stable oxide layer on the metal surface is plasma electrolytic oxidation [165].

Surface coating of dental implants is used to enhance osseointegration and reduce corrosion [166]. The protective layer on the surface may be formed in different ways, including sol-gel surface coating, physical or chemical vapor deposition, and plasma spraying [89].

Plasma immersion ion implantation is used for surface modification of dental implants with different ions to enhance biocompatibility [167,168].

Layer-by-layer electrostatic self-assembly is also a promising method of titanium surface modification based on interactions of different electrolytes. With this method, new properties of the implant surface may be achieved, such as bone healing stimulation, antibacterial effect, corrosion resistance, etc. [169].

Short-term clinical trials showed faster osseointegration of dental implants after different surface treatments [170,171]. However, it should be noted that there is a lack of clinical evidence regarding the long-term effects of dental implant surface modification and the potential impact on bio-tribocorrosion has not been studied sufficiently [172].

### 5.2. Clinical Recommendations for Treatment Planning

The treatment plan should be developed considering the information about the patient’s general medical history. Given that patients having gastrointestinal problems, endocrine diseases, or autoimmunity may have an increased risk of dental alloys’ side effects, physician consultation is recommended. If possible, non-metal prosthetic appliances should be used. If not, one should avoid using dental alloys with low corrosion resistance and galvanic coupling of dental alloys. Furthermore, a history of any allergic reactions to metals or metal intolerance such as contact allergy to jewelry is an indication for a complete allergy examination, including patch-testing and blood tests [173]. Given that the patch-test may cause false-positive reactions, an optimized lymphocyte transformation test (LTT) should be used to confirm the diagnosis of metal hypersensitivity [63].

In the case of pre-existing oral lesions such as leukoplakia, oral lichen planus, or recurrent aphthous stomatitis, additional allergy tests are required before the treatment. If possible, non-metal appliances or noble dental alloys should be preferred. 

If any metallic appliances are already present in the oral cavity, it would be useful to know their exact composition to avoid galvanic coupling with new dental alloys. Anyway, the method of choice is complete prosthetic rehabilitation using a dental alloy with high corrosion resistance. 

### 5.3. Recommendations for Patients with Metallic Prosthetic or Orthodontic Appliances

To minimize the risk of adverse events, it would be useful to avoid factors increasing corrosion of dental alloys, for example: smoking and hard alcohol consumption, use of ethanol-based mouth rinses and fluoride-containing oral hygiene products, use of abrasive toothpastes, and regular intake of acidic foods and beverages.

### 5.4. Recommendations for Patients with Oral and/or Systemic Symptoms, Potentially Associated with Dental Alloys

As mentioned above, the most common clinical manifestations of adverse effects associated with dental alloys include allergic reactions, mouth burning, and white oral lesions. Regarding hypersensitivity, it may be diagnosed by the use of skin or blood tests. Nevertheless, the diagnosis of oral galvanism remains a complicated issue since, to date, there is no standardized method for the determination of intra-oral electric currents. Several clinical studies reported intra-oral galvanic current assessment with the zero-resistance ammeter [41,174] or with a specialized dental voltmeter/ammeter [175]. It should also be taken into account that the current strongly depends on the electrical resistance of the intra-oral medium, which is very changeable. Another method for galvanism diagnosis is the measurement of electrochemical potentials of metallic appliances and the determination of potential differences between coupling dental alloys [11,53,81]. However, these methods are not widely used in routine dental practice, and dentists commonly opt for the replacement of metallic appliances according to patients’ complaints and clinical symptoms.

**Figure 4:**
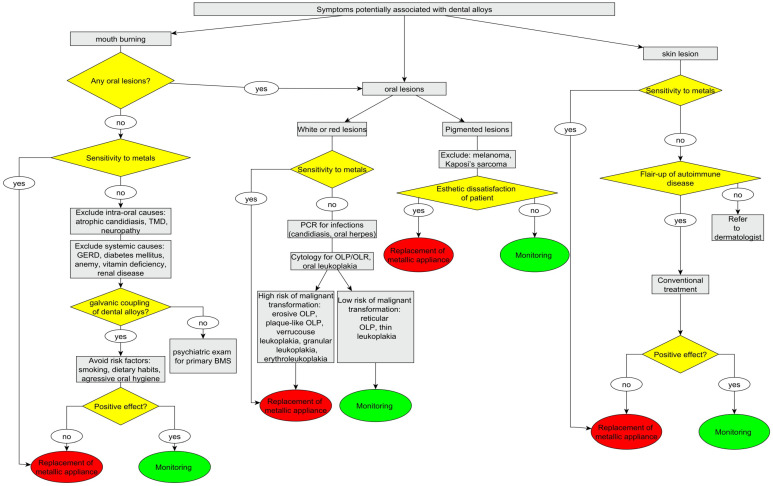
Decision tree for diagnosis and management of conditions potentially associated with dental alloys.

Replacement of dental alloys may be recommended in the following cases: flare-up of autoimmune diseases; severe mouth burning that appears after placement of metallic appliances; positive path-test or blood test to components of dental alloys; and the appearance of potentially malignant oral lesions [58]. As the clinical picture of dental alloys’ side effects is not specific, other diseases with similar symptoms should be excluded before replacement of metallic appliance, and all the other possible causes should be eliminated (Figure 4). 

## 6. Conclusions

The issue of adverse events associated with dental alloys remains due to the extensive use of different metallic appliances in dentistry. The side effects of dental alloys may have multiple local and systemic manifestations, which may affect the quality of life and complicate the diagnosis. Since the replacement of dental implants and prosthetic appliances is a time-consuming and expensive procedure, dentists should focus on prevention rather than on diagnosis and treatment of adverse events. Therefore, understanding the basic pathogenesis mechanisms and risk factors of dental alloys’ side effects is necessary to provide an optimal treatment plan and recommendations for patients with metallic oral appliances.

## Figures and Tables

**Table 1:** Factors increasing the risk of adverse effects associated with dental alloys.

	Risk Factors	Mechanism of Action
Local factors	Use of dental alloys with low corrosion resistance	Increased release of metal ions
	Galvanic coupling of different dental alloys in the oral cavity	Increased release of metal ions, chronic irritation of the oral mucosa with the direct current
	Poor oral hygiene	Increased corrosion due to acidogenic flora activity
	Multiple caries Periodontal disease	Decreased salivary pH and increased corrosion
	Fluoride-containing oral hygiene products	The increase in galvanic current and corrosion
	Ethanol-based oral rinses	The increase in galvanic current and corrosion
	Abrasive toothpastes	Surface degradation of dental alloys
Habitual factors	Smoking and hard alcohol consumption	Decreased resistance and healing potential of the oral mucosa
	Regular intake of acidic foods and drinks	The increase in galvanic current and corrosion
Systemic factors	Radiotherapy	Decreased resistance and healing potential of the oral mucosa, decreased salivary pH
	Gastro-intestinal reflux disease	The increase in galvanic current and corrosion
	Post-menopausal period in women Diabetes mellitus Thyroid hypofunction	Decreased salivary pH, altered taste, increased sensitivity of the oral mucosa
	Renal disease	Increased electroconductivity of the whole saliva
	Autoimmune diseases	Potential hypersensitivity to metals

## Data Availability

Not applicable.
